# MexXY Multidrug Efflux System Is More Frequently Overexpressed in Ciprofloxacin Resistant French Clinical Isolates Compared to Hospital Environment Ones

**DOI:** 10.3389/fmicb.2019.00366

**Published:** 2019-02-26

**Authors:** Cristiano Serra, Bakhta Bouharkat, Aicha Tir Touil-Meddah, Stéphanie Guénin, Catherine Mullié

**Affiliations:** ^1^Laboratoire des Agents Infectieux, Resistance et Chimiothérapie – EA 4294, UFR de Pharmacie, Université de Picardie Jules Verne, Amiens, France; ^2^Laboratoire de Bioconversion, Génie Microbiologique et Sécurité Sanitaire N°145, Faculté des Sciences de la Nature et de la Vie, Université Mustapha Stambouli, Mascara, Algeria; ^3^Centre de Ressources Régionales en Biologie Moléculaire, UFR des Sciences, Université de Picardie Jules Verne, Amiens, France; ^4^Laboratoire d’Hygiène Hospitalière, Centre Hospitalier Universitaire Amiens-Picardie, Amiens, France

**Keywords:** *Pseudomonas aeruginosa*, efflux pump, overexpression, fluoroquinolone, resistance, environment

## Abstract

Modulation of the membrane permeability through a decrease in porin-mediated antibiotic entry and/or an increase in antibiotic efflux is one of the resistance mechanisms to antibiotics evolved by Gram-negative bacteria. To assess whether the outer membrane porin OprD and Resistance-Nodulation-Division (RND) efflux pumps were similarly expressed in 33 ciprofloxacin-resistant clinical strains of *Pseudomonas aeruginosa* and in 30 non-clinical strains originating from the hospital environment (mainly waterborne *Pseudomonas aeruginosa*), the expression of *oprD*, *mexB*, *mexF*, and *mexY* genes was investigated. Overall, the expression of *oprD* was not detected by RT-qPCR in 14 (22%) strains and underexpressed in 35 (56%) more. No significant difference in *oprD* expression was detected between clinical and non-clinical strains. As for efflux pumps, 23 (70%) of the clinical strains overexpressed at least one of the tested RND genes. Overexpression of *mexB*, *mexF* and *mexY* was detected in 27, 12, and 45% of the clinical strains, respectively. In the 30 non-clinical strains, no overexpression could be found for *mexB*, *mexF*, or *mexY.* On the contrary, a global underexpression of the tested efflux pump genes was recorded. In both clinical and environmental strains, a positive correlation was found between the expressions of *oprD* and *mexB*. Similarly, the expressions of *oprD* and *mexF* were positively correlated. This result contrasts with the inverse correlation between both MexAB-OprM/MexEF-OprN and OprD previously described in carbapenem-resistant *P. aeruginosa* strains. The only positive correlation between phenotypic ciprofloxacin minimum inhibitory concentrations (MICs) and the expression of efflux pump gene was witnessed with *mexY* (analysis on pooled results for clinical and environmental strains). However, in clinical strains, no statistically significant link could be found between the degree of reduction in ciprofloxacin MICs witnessed with Phenylalanine-Arginine β-naphthylamide (PAβN) and the expression of any of the 3 RND genes tested.

## Introduction

*Pseudomonas aeruginosa* is a Gram-negative ubiquitous microorganism. It can be found in various environmental ecological niches (e.g., water, soil, and plants) but can also infect humans (Botzenhart and Döring, 1993). It more specifically affects individuals with impaired defenses such as patients with severe burns, cancer or cystic fibrosis (Gellatly and Hancock, 2013). *P. aeruginosa* is innately resistant to a large number of commercially available antibiotics (Poole, 2011) and, like most other species of bacteria, has acquired a wide array of resistance mechanisms, tremendously complicating the clinical handling of *P. aeruginosa* infections and leading to the emergence of so-called pan resistant strains (Bonomo and Szabo, 2006; Poole, 2011). Modification of the membrane permeability is one of the mechanisms by which Gram-negative bacteria can decrease their susceptibility to antibiotics, by a reduction in the antibiotic entry through outer membrane porins and/or an increase in antibiotic efflux through efflux pumps (EPs). For example, in *P. aeruginosa*, mutations in the porin OprD can lead to the resistance to carbapenems (Xia et al., 2016; Del Barrio-Tofiño et al., 2017). Overexpression of EPs belonging to the Resistance-Nodulation-Division (RND) family, i.e., MexAB-OprM, MexCD-OprJ, MexEF-OprN and MexXY-OprM, has also been pointed out as a resistance mechanism toward various antibiotic families such as β-lactams, aminoglycosides or fluoroquinolones (FQ) (Poole, 2011). Additionally, an inverse association between the expressions of *oprD* and genes encoding RND EPs has been pointed out in carbapenem-resistant *P.aeruginosa* strains (Köhler et al., 1999; Ikonomidis et al., 2008; Xavier et al., 2010; Lee and Ko, 2012). It was demonstrated that MexT, a positive regulator of *mexEF-OprN* expression, negatively regulates the expression of OprD in carbapenem-resistant strains (Köhler et al., 1999).

RND EPs are constituted by three proteins: the first one is acting as an outer membrane channel [a protein belonging to the outer membrane factor (OMF) family, e.g., OprM], the pump itself is embedded in the cytoplasmic membrane (the actual RND protein of the pump, e.g., MexB) and, in the periplasmic space, a linker protein is joining the first two proteins [this protein is also referred to as the major facilitator protein (MFP), e.g., MexA] (Piddock, 2006). These pumps are involved in *P. aeruginosa* physiological functions such as quorum-sensing (Minagawa et al., 2012), biofilm formation (Alav et al., 2018) and virulence factors (Blair and Piddock, 2009; El-Shaer et al., 2016). Expression and overexpression have been described when a mutation occurs in the repressor/activator gene sequences commanding the operons encoding EPs (Poole, 2011; López-Causapé et al., 2018). Useful details on the systems regulating EP expression in *P. aeruginosa* can be found in reviews such as Poole (2011) and Morita et al. (2012). The aim of the present work was to assess the expression of OprD and RND EPs was different in clinical strains resistant to ciprofloxacin (CIP), a molecule belonging to the fluoroquinolone (FQ) family and in *P. aeruginosa* strains retrieved from non-clinical (mainly environmental) samples isolated from a hospital background. Phenotypic resistance to CIP was chosen as a tracker for the overexpression of EPs as FQs are good substrates for the four major RND pumps found in *P. aeruginosa* (Poole, 2011). Assessing whether the CIP phenotypic resistance profile of *P. aeruginosa* strains could be related to the expression of one or several genes encoding for the major RND EPs could be helpful to evaluate the *in vitro* efficacy of new potential efflux pump inhibitors on relevant clinical strains resistant to this molecule.

## Materials and Methods

### Bacterial Strains

Thirty-three clinical strains of *P. aeruginosa* resistant to ciprofloxacin (CIP) isolated from 33 patients of Amiens University Hospital (France) were collected over a 5-month period and included in this study. The selection of these clinical strains was made by screening the results of all in-coming clinical samples (no specific unit or ward targeted) and retaining *P. aeruginosa* strains detected as resistant to CIP (CIP-R) by the disk diffusion method used routinely. If multiple samples yielded a ciprofloxacin-resistant strain for a given subject, only one isolate was included in the panel of strains. Resistance was confirmed through minimum inhibitory concentration (MIC) determination. MICs were determined over a range of 0.0625 to 128 μg/mL on 96-microwell plates using the broth microdilution technique in cation-adjusted Mueller-Hinton (CLSI, 2015). Resistance to CIP was determined for MICs of 1 μg/mL and above, according to EUCAST latest breakpoints (European Committee on Antimicrobial Susceptibility Testing [EUCAST], 2018).

Environmental strains consisted of *P. aeruginosa* strains previously isolated from non-clinical samples and retrieved from the culture collection of Amiens University Hospital as well as strains prospectively obtained over a 2-year period (May 2016–May 2018) from water samples systematically collected as part of the program for water quality monitoring in Amiens University Hospital and surrounding hospitals. This program includes the detection of *P. aeruginosa* on a cetrimide agar (Biomérieux, Marcy-l’Etoile, France) according to the current ISO guidelines (EN ISO 16266:2008, 2008) and is based on a bi-annual sampling of critical control points of the hospital water supply system. Overall, 208 critical control points have been identified through an internal risk analysis and are sampled routinely.

A target number of 30 strains for each group (clinical and non-clinical) was set at the beginning of the study. While this target was easily met for clinical strains, the prospective collection of environmental strains originating from the water quality monitoring program was slow going. As only five *P. aeruginosa* strains had been isolated at the end of 2016, it was decided to include *P. aeruginosa* strains previously isolated from the environment and as well as non-clinical samples that had been kept in the laboratory collection in addition to the prospective collection of such strains. A total of 30 non-clinical strains was reached in May 2018.

Two strains were considered for the role of reference (calibrator) in RT-qPCR assays: a strain (AM19) from the same clinical background as CIP-R strains but susceptible to CIP and *P. aeruginosa* DSM (Deutsche Sammlung für Mikroorganismen und Zellkulturen, Braunschweig, Germany) 1117/ATCC (American Type Culture Collection) 27853, a reference *P. aeruginosa* strain used as quality control in antimicrobial susceptibility testing (Fang et al., 2012). All strains were kept frozen at -80°C on microbeads (WVR International SAS, Fontenay-sous-Bois, France) until use.

### Ciprofloxacin Minimum Inhibitory Concentration (MIC) and Phenotypic Efflux Determination

PAβN (Sigma-Aldrich, Saint-Quentin-Fallavier, France) has been described as an efflux pump inhibitor (EPI) affecting MexAB-OprM, MexCD-OprJ and MexEF-OprN EPs (Lomovskaya et al., 2001) and has also been shown to reduce levofloxacin MIC in a *P. aeruginosa* strain with an increased MexXY activity (Mao et al., 2001). This molecule was subsequently used as such to phenotype-wise determine the prevalence of efflux pump-mediated resistance to fluoroquinolones in *P. aeruginosa.* It was added to the standard MIC determination procedure at a fixed final concentration of 50 μg/mL (Sonnet et al., 2012). MIC determination for PAβN was also carried out for all strains over concentrations ranging from 0.1 to 200 μg/mL, to ascertain that the 50 μg/mL concentration used for EP inhibition was not inhibitory in itself. The phenotypic efflux was deemed significant when the MIC determined in the presence of PAβN was at least fourfold lower than the MIC witnessed in its absence (Piddock, 2006; Sonnet et al., 2012), leading to a calculated phenotypic efflux factor (MIC without PAβN/MIC with PAβN) of four and above.

### RNA Extraction and Reverse Transcription

Strains were cultured in cation adjusted Mueller-Hinton Broth (Merck, Darmstadt, Germany) for 18 to 24 h at 37°C up to the late exponential phase prior to EP expression determination. Total RNA extraction was carried out using the Qiagen RNeasy purification kit with the addition of RNAprotect Bacteria Reagent (Qiagen, Courtaboeuf, France). Briefly, 1 mL of culture medium was centrifuged (8000 *g*, 5 min), the bacterial pellet resuspended in 1 mL of RNAprotect reagent and left to incubate at room temperature for 5 min. After decantation of the RNAprotect reagent (8000 *g*, 10 min), 200 μL of Tris 30 mM-EDTA 1 mM (pH 8.0) buffer containing lysozyme (15 mg/mL) and proteinase K (0.56 mg per strain) were added and incubated for 10 min at room temperature to ensure a complete bacterial cell wall lysis. Further precipitation and purification of nucleic acids were performed according to the manufacturer’s recommendations and the final elution step carried out using 50 μL RNase-free water. After checking the RNA extraction quality on a 1% agarose gel and measuring the RNA content (Nanodrop, ThermoFisher Scientific, France), RNA extracts were stored at -20°C until further use.

Prior to cDNA synthesis, genomic DNA was removed from 1 μg of total RNA using the gDNA wipeout buffer included in the Quantitect^®^ Reverse Transcription kit (Qiagen). The reverse transcription was performed under a volume of 20 μL including 14 μL of template RNA (extract concentrations adjusted to contain 1 μg of RNA), 1 μL of reverse transcription master mix, 4 μL RT buffer 5x (containing dNTPs and Mg^2+^) and 1 μL of RT primer mix containing random hexamers. Reverse transcription was performed in a Veriti PCR Thermal Cycler (Applied Biosystems, France) for 30 min at 42°C followed by a 3 min incubation at 95°C to inactivate the reverse transcriptase.

All reactions including RNA handling were carried out on ice.

### Real-Time PCR Assay

Primers used in this study were designed using the Primer3 software available online^1^ and are listed in Table 1. As the genes encoding EP components are organized in operons (Poole et al., 1993; Morita et al., 2001), this work focused on the expression of *oprD* and *mexB*, *mexF*, *mexY* genes encoding the actual efflux pump protein of the three main RND EPs found in *P. aeruginosa.*

**Table 1:** Primers used in this study.

Gene	Amplicon size (bp)	Primer sequence (5′→ 3′) FWD/REV
*mexB*	167	CAACATCCAGGACCCACTCT
		AGGAAATCTGCACGTTCTGC
*mexF*	163	TGTACGCGAACGACTTCAAC
		GAGGTGTCGCTGACCTTGAT
*mexY*	159	TCAGGCCGACCTTGAAGTAG
		TCTCGGTGTTGATCGTGTTC
*oprD*	157	GCCGAAGCCGATATAATCAA
		CATCTACCGCACAAACGATG
*rpsL*	163	TACTTCGAACGACCCTGCTT
		TTTCCTCGTACATCGGTGGT


Normalization of expression results was carried out using *rpsL* (Llanes et al., 2004; Yoneda et al., 2005).

A Lightcycler 480 (Roche Diagnostics, Meylan, France) was used for all quantitative PCRs. All PCR amplification reactions were performed in 384-well-plates under a 10 μL final volume containing 2.5 μL of diluted (1:20) template cDNA, 1 μL of each primer (corresponding to a final concentration of 0.5 μM), 5 μL of Quantitect SYBR Green PCR Master Mix (including MgCl_2_ to reach to final concentration of 2.5 mM) (Qiagen) and 0.5 μL RNase/DNase free water (Qiagen).

The cycling program was set as follows : (1) activation : 1 cycle at 95°C for 15 min, (2) amplification : 45 cycles including a 15-s denaturation at 95°C, a 25-s annealing at 60°C and a 15-s elongation at 72°C, and (3) melting curve : 1 cycle including 5 s at 95°C, 1 min at 65°C and a final increase at 97°C with a transition rate of 0.11°C/s.

Each reaction was carried out in duplicate and the experiment was repeated on two different sets of RNA extracts (biological replicate).

### Evaluation of Real-Time PCR Results

Relative standard curves describing the PCR efficiency (E) for each primer pair were generated for each amplicon (Larionov et al., 2005).

Prior to any other analysis, melting curves were checked for the presence of primer dimers and other artifacts. The relative expression between the target and reference genes, was calculated using the formula (*E*_*goi*^∧^(Δ*Ct*,*goi*))/(*E*_*ref*^∧^(Δ*Ct*,*ref*)), where *E* stands for the PCR efficiency factor, *goi* for gene of interest and *ref* for reference.

Results were subsequently expressed as Normalized Calibrated Ratios (NCRs).

### Statistical Analysis

Differences in the expression of each gene of interest were tested using the single sample *t*-test vs. cut-off values of 0.5 for underexpression and 2 for overexpression, respectively (Tomás et al., 2010). Differences in gene detection and expressions between clinical and non-clinical/environmental strains were assessed using Fisher’s exact test.

Correlations between gene expressions, MICs with and without PAβN as well as with the MIC reduction factor were tested using Spearman’s correlation coefficient.

Statistical significance was inferred for *p*-values below 0.05.

The statistical analysis was performed using VassarStats website^2^ and XLstat 2015 software (Addinsoft, France).

### Ethics Statement

As strains were isolated from clinical samples routinely prescribed to patients by the medical staff in charge and as no specific sampling in order to collect *P. aeruginosa* strains was performed, no evaluation by the local ethics committee was sought. Additionally, written informed consent from the patients was not needed as this work was of a retrospective nature and as no information that could be linked to patients was included.

## Results and Discussion

### Strain Collection, CIP MICs, and Phenotypic Efflux Factors

Tables 2, 3 summarize the origins, MICs with and without PAβN and the resulting calculated phenotypic efflux factor for each of the 33 CIP-R clinical strains and 30 non-clinical/environmental strains evaluated in this work, respectively. Only 3 (10%) these non-clinical/environmental strains were classified as resistant to CIP with a MIC of 1 μg/mL according to EUCAST 2018 standards (European Committee on Antimicrobial Susceptibility Testing [EUCAST], 2018).

**Table 2:** Characteristics of the clinical strains included in this study, classified by increasing Ciprofloxacin (CIP) MIC.

Strain	Clinical origin	CIP MIC (μg mL^-1^)		Phenotypic efflux factor^a^	RND pump gene overexpressed
		PAβN -	PAβN +		
AM19	Urinary tract	<0.0625	ND^b^	ND	Reference strain
DSM1117	Blood culture	0.125	ND	ND	*mexB*
AM126	Rectal swab	2	0.25	8	*mexB*
AM3	Blood culture	2	0.0625	32	*mexB*
AM60	Tracheal	2	0.0625	32	*mexB*
AM99	Sputum	2	0.0625	32	*mexY*
AM17	Sputum	4	1	4	*mexF*
AM74	Skin wound	4	0.5	8	*mexB, mexY*
AM33	Sinus	4	0.0625	64	*mexB*
AM66	Peritoneum	4	0.0625	64	None
AM83	Urinary tract	8	2	4	*mexY*
AM100	Ear	8	4	2	None
AM131	Rectal swab	8	2	4	None
AM32	Ear	16	4	4	None
AM44	Urinary tract	16	4	4	*mexY*
AM50	Urinary tract	16	4	4	*mexY*
AM52	Urinary tract	16	2	8	*mexY*
AM56	Ear	16	2	8	*mexB*
AM86	Tracheal	16	2	8	*mexB*
AM88	Urinary tract	16	2	8	*mexY*
AM115	Urinary tract	16	2	8	*mexB*
AM127	Rectal swab	16	2	8	None
AM1	Blood culture	16	1	16	None
AM27	Tracheal	16	1	16	*mexF, mexY*
AM85	Sputum	16	1	16	*mexB, mexF, mexY*
AM10	Urinary tract	32	8	4	*mexY*
AM42	Urinary tract	32	8	4	*mexF,mexY*
AM130	Rectal swab	32	8	4	None
AM13	Urinary tract	32	4	8	None
AM110	Sputum	32	4	8	*mexY*
AM128	Rectal swab	64	16	4	None
AM129	Rectal swab	64	16	4	None
AM69	Tracheal	64	16	4	*mexY*
AM113	Prepuce	64	8	8	*mexY*
AM58	Urinary tract	128	64	2	*mexY*


*^a^MIC without PAβN/MIC with PAβN. ^b^Not determined.*

**Table 3:** Characteristics of non-clinical/environmental strains included in this study, classified by increasing Ciprofloxacin (CIP) MIC.

Strain	Environmental origin, town	Sampling date	CIP MIC (μg mL^-1^)		Phenotypic efflux factor	RND pump gene overexpressed
			PAβN -	PAβN +		
ENV1	Water, Amiens	2016	<0.0625	ND	–	None
ENV2	Water, Amiens	2016	<0.0625	ND	–	None
ENV3	Human milk, Amiens	2016	<0.0625	ND	–	None
ENV8	Siphon, Amiens	2016	<0.0625	ND	–	None
ENV9	Siphon, Amiens	2016	<0.0625	ND	–	None
ENV12	Water, Amiens	1995	<0.0625	ND	–	None
ENV18	Water, Amiens	1997	<0.0625	ND	–	None
ENV19	Water, Amiens	1997	<0.0625	ND	–	None
ENV20	Water, Amiens	1997	<0.0625	ND	–	None
ENV4	Siphon, Amiens	2016	0.125	ND	–	None
ENV5	Endoscope, Amiens	2016	0.125	ND	–	None
ENV6	Water, Amiens	2016	0.125	ND	–	None
ENV7	Water, Amiens	2016	0.125	ND	–	None
ENV23	Endoscope, Amiens	2017	0.125	ND	–	None
ENV24	Water, Amiens	2017	0.125	ND	–	None
ENV25	Water, Albert	2017	0.125	ND	–	None
ENV27	Water, Amiens	2018	0.125	ND	–	None
ENV30	Water, Amiens	2018	0.125	ND	–	None
ENV13	Irrigation medical device, Amiens	1995	0.125	ND	–	None
ENV10	Siphon, Amiens	2016	0.25	ND	–	None
ENV26	Water, Amiens	2018	0.25	ND	–	None
ENV28	Water, Amiens	2018	0.25	ND	–	None
ENV29	Water, Amiens	2018	0.25	ND	–	None
ENV11	Water, Doullens	2016	0.5	<0.0625	>8	None
ENV14	Water, Amiens	1997	0.5	<0.0625	>8	None
ENV15	Water, Amiens	1997	0.5	<0.0625	>8	None
ENV16	Water, Amiens	1997	0.5	<0.0625	>8	None
ENV17	Siphon, Amiens	1997	1	<0.0625	>16	None
ENV21	Water, Amiens	1999	1	<0.0625	>16	None
ENV22	Water, Amiens	1999	1	<0.0625	>16	None


Most (31/33, ∼94%) CIP-R clinical strains displayed a phenotypic efflux factor of four or above (Table 2). All threeCIP-R environmental strains had a phenotypic efflux factor above 16 (Table 3). The lower phenotypic efflux factor witnessed in clinical strains could be explained by a resistance only partly mediated through an increased efflux. Indeed, the higher MICs (up to 128 μg/mL) in clinical strains might be linked to cumulative mutations in CIP targets, DNA gyrase and topoisomerase IV proteins, as described previously (Bruchmann et al., 2013; Rehman et al., 2019). In the environmental strains, efflux could be the sole mechanism supporting the rise of the MIC up to 1 μg/mL. Determination of QRDR mutation frequencies in the environmental strains included in this study would have reinforced this hypothesis. Nevertheless, the study by Bruchmann et al. (2013) has already shown that most QRDR mutations lead to CIP MICs equal or superior to 1 μg/mL while strains only overexpressing EPs displayed a maximum MIC of 2 μg/mL. Our results are therefore in line with this report. Also, they are in agreement with a theory stating that the overexpression of EPs could act as a first-step mutation, leading to subsequent ones in DNA gyrase and topoisomerase IV proteins and higher levels of CIP resistance (Köhler et al., 1997; Vila and Martínez, 2008).

### EP Gene Overexpression

First, gene expression was compared between the two CIP-susceptible strains included in this work as possible calibrators for NCR calculations. *P. aeruginosa* DSM1117, a clinical strain from a culture collection used in antibiotic testing (European Committee on Antimicrobial Susceptibility Testing [EUCAST], 2018), displayed a significant overexpression of *mexB* and underexpression of *mexY* as compared to the clinical strain AM 19 (Supplementary Table S1). The reasons for choosing *P. aeruginosa* AM19 over *P. aeruginosa* DSM1117 as a calibrator in our calculations were that (i) AM19 came from a similar background as the other strains enrolled in this study and (ii) *oprD* expression was not detected in *P. aeruginosa* DSM1117 and would therefore not allow the calculation of NCRs for this gene. Thereafter, individual NCRs were calculated as mean ± sem (standard error of the mean) for each strain, using *P. aeruginosa* AM19 as calibrator (Supplementary Table S1). If *P. aeruginosa* DSM1117 had been chosen as a calibrator, the potential differences in NCRs results for EP genes would have been (i) a greater frequency of overexpression for *mexY* gene in clinical strains, as it was underexpressed in *P. aeruginosa* DSM1117 as compared to *P. aeruginosa* AM19, and (ii) a lower frequency of *mexB* overexpression in clinical strains (Supplementary Table S1). As for non-clinical strains, the results would have remained similar, EP gene expressions being overall low among these strains.

At least one EP was overexpressed in 23 (70%) out of the 33 clinical strains included in this work (Table 2). One could argue that the mRNA expression level for a given gene is not directly related to the actual amount of protein produced by the bacteria. However, Yoneda et al. (2005) previously showed a good correlation between the amounts of MexY and MexB proteins and their respective mRNA expressions.

Hocquet et al. (2007) reported a simultaneous overexpression of MexAB-OprM and MexXY in a set of ticarcillin-resistant clinical strains originating from 15 French hospitals. No such joint overexpression was witnessed for the CIP-R strains in this study. Only three clinical strains overexpressed two EPs simultaneously (*mexF/mexY* for two strains and *mexB/mexY* for one). Additionally, a single clinical strain overexpressed all three RND genes tested in this work (Table 2).

A high prevalence of *mexY* overexpression was witnessed in the series of clinical strains (Table 4). MexXY expression has been shown to be regulated by repressor MexZ but also by an independent two-component system, ParRS (Morita et al., 2012). The frequent overexpression of *mexY* witnessed here might therefore be linked to mutation(s) in this repressor sequence and/or the triggering of ParRS system. MexAB-OprM appeared to be either up- or down-regulated (Table 4). Similar results were reported in multidrug-resistant strains of *P. aeruginosa* in Bulgaria (Vatcheva-Dobrevska et al., 2013). Only four clinical strains were found to overexpress MexEF-OprN (Table 3), ruling out its role as a major contributor to CIP efflux mediated resistance.

**Table 4:** Efflux pump genes and *oprD* detection and expression in clinical (*n* = 33) and non-clinical (*n* = 30) *Pseudomonas aeruginosa* strains isolated in this study.

	*mexB*	*mexF*	*mexY*	*oprD*
**Positive RT-qPCR detection**				
Overall	54 (86)^a^	52 (83)	57 (90)	29 (46)
Clinical	33 (100)^∗^	32 (97)^∗∗^	33 (100)^∗∗^	15 (45)
Non-clinical	21 (70)^∗^	20 (67)^∗∗^	24 (80)^∗∗^	14 (47)
**Overexpression**				
Overall	9 (14)	4 (6)	15 (24)	0 (0)
Clinical	9 (27)^†^	4 (12)	15 (45)^∗^	0 (0)
Non-clinical	0 (0)^†^	0 (0)	0 (0)^∗^	0 (0)
**Underexpression**				
Overall	39 (62)	46 (73)	35 (56)	49 (78)
Clinical	11 (33)^∗^	18 (55)^†^	5 (15)^∗^	23 (70)
Non-clinical	28 (93)^∗^	28 (93)^†^	30 (100)^∗^	26 (87)


*RT-qPCR detection was considered positive for a given gene when cycle thresholds and subsequent Normalized Calibrated Ratios could be calculated from the experiments.*

*^a^Number of strains (%).*

*^∗^Significant difference between clinical and non-clinical strains (p < 0.0001, Fisher’s exact test).*

*^∗∗^Significant difference between clinical and non-clinical strains (p < 0.001, Fisher’s exact test).*

*^†^Significant difference between clinical and non-clinical strains (p < 0.01, Fisher’s exact test).*

A positive correlation was found between *mexB* and *mexF* expressions (Pearson’s *r* = 0.557, *p* = 0.001). This result is at odds with regulation pathways previously described. Indeed, in addition to mutations in its negative regulators MexR, NalC, and NalD, the overexpression of MexB has been linked with quorum-sensing signaling (Maseda et al., 2004; Poole, 2011). Quorum-sensing autoinducers were shown to be implied in an inverse regulation of *mexB* and *mexF* expressions along with MexR and MexT regulators (Maseda et al., 2004). However, the interplay between *mexF* and *mexB* might not be so straightforward as (i) other reports also describe a simultaneous overexpression of MexAB-OprM and MexEF-OprN (Tomás et al., 2010) and (ii) other regulation pathways might be implied such as those including MexS, another regulator of MexEF-OprN (Uwate et al., 2013).

The results for MexCD-OprJ were not included in this paper, as preliminary results for *mexD* expression in clinical strains showed that none of the strains displayed changes in the expression of this gene.

In contrast to clinical strains, none of the environmental strains overexpressed any EP (Tables 3, 4 and Supplmentary Table S1). Expression of RND EP genes in environmental *P. aeruginosa* strains is seldom reported in the literature (Braz et al., 2016; Maravić et al., 2018). These studies showed that MexAB-OprM overexpression was linked to resistance to aztreonam and other β-lactams in *P. aeruginosa* strains isolated from agricultural soils and marine shellfish, respectively. They showed environmental strains can display features predisposing them to antibiotic resistance, which could, in turn, pose a threat to human and environmental health. This was not the case for the environmental strains included in this study.

### Expression of *oprD* and Correlations the Expression of EP Genes

Only about 50% of the tested strains displayed detectable levels of *oprD* mRNA (Table 4). No significant difference was found in the distribution of *oprD* expression profiles between clinical and non-clinical strains (Table 4). Positive correlations were found with both *mexB* and *mexF* expressions (i) in clinical strains: *mexB/oprD* (Pearson’s *r* = 0.417, *p* = 0.016) and *mexF/oprD* (*r* = 0.476, *p* = 0.005) and (ii) in environmental strains: *mexB/oprD* (Pearson’s *r* = 0.494, *p* = 0.006) and *mexF/oprD* (*r* = 0.649, *p* < 0.001, respectively).

An *oprD* repression has previously been reported as concomitant to MexAB-OprM, MexXY-OprM and MexEF-OprN overexpression in *P. aeruginosa* strains resistant to various β-lactams (Köhler et al., 1999; Ikonomidis et al., 2008; Xavier et al., 2010). Additionally, the link between *mexB* and *oprD* expressions in β-lactam resistant strains has been suggested to reflect an external pressure, such as antibiotic exposure, affecting both systems (Horna et al., 2018). However, the statistical significance of a possible correlation between these EPs and OprD expression remains to be ascertained in strains selected on the basis of other antibiotic resistances. For instance, in this study, no repression of *oprD* expression could be witnessed either in the CIP-R clinical *P. aeruginosa* strains when EPs are overexpressed.

### Correlation Between the Expression of EP Genes, CIP MICs and the Phenotypic Efflux Factor

As the growth stage has been shown to influence the expression levels of EP genes (Mesaros et al., 2007), *P. aeruginosa* strains were all grown up to the late exponential phase prior to RNA extraction to minimize variations and standardize culture conditions. This step insured the relevance of the correlation drawn between MICs and gene expression levels as MICs are classically determined after an 18 to 24-h incubation. CIP was used as a marker to detect efflux-mediated resistance to antibiotics as it has been shown to be a substrate for various EPs (e.g., NorA for *Staphylococcus aureus*, OqxAB for *Klebsiella pneumoniae*, AcrAB-TolC for *Escherichia coli*, AdeABC for *Acinetobacter baumannii*), including *P. aeruginosa* RND ones (Poole, 2005). When clinical and non-clinical strains were pooled together, CIP MICs were loosely correlated with *mexY* expression (Pearson’s *r* = 0.400, *p* = 0.001). When the statistical analysis was held on clinical and environmental strains separately, no correlation between the expression of EP genes and MICs could be pointed out. For CIP-R clinical strains, a likely explanation for this lack of correlation is the presence of co-existing mutations in gyrase and topoisomerase IV proteins (López-Causapé et al., 2018). Additionally, other parameters might also contribute to this lack of correlation such as resistance to aminoglycosides and/or β-lactams. Indeed, these antibiotic families are also known to be related to EPs’ expression (Poole, 2011). It would therefore be interesting to further evaluate aminoglycosides and carbapenem resistances in these strains and the presence/absence of correlation with their expression of EPs.

To address a possible correlation between the reduction in CIP MICs in the presence of PAβN and EP expression, non-clinical strains were removed from the analysis as their initial low CIP MICs would not allow for a significant reduction (Table 3). Hence, for clinical strains, no statistically significant correlations between EP expression, the MIC reduction or the phenotypic efflux were detected. Therefore, CIP MICs in the presence of PAβN are not predictive of the overexpression of RND EPs in *P. aeruginosa.* Interfering factors such as a variable expression for other EPs and/or a decrease in MIC with PAβN not entirely linked with efflux inhibition could explain this lack of correlation. Lamers et al. (2013) indeed put forward that PAβN not only blocks EPs but also permeabilizes the outer membrane. In these conditions, entry of large antibiotics such as vancomycin and macrolides are facilitated. Such might also be the case for CIP (even though it is a smaller molecule which would not require outer membrane permeabilization to enter), hence further contributing to a reduction in apparent MICs. This might also explain why a high phenotypic efflux factor could be calculated for the three CIP-R environmental when no RND EP overexpression was detected in those strains. Nevertheless, this work enabled the description of clinical strains with various profiles of RND EP overexpression that will be used as laboratory tools for the evaluation of newly synthesized candidates for efflux pump inhibition.

To gain further insight in the relationships between the expression of RND EPs and phenotypic resistance to CIP in environmental strains, the collection of additional strains displaying such a resistance would be of interest as well as collecting strains from different hospitals to broaden the conclusions drawn from the results of this first study. It would also be interesting to investigate whether additional CIP susceptible clinical strains share the expression profile RND EPs’ of their resistant counterparts or if their profile is closer to the one of environmental strains.

## Conclusion

The results obtained on the 33 clinical strains pertaining to this study showed that the main contributor to efflux-mediated CIP resistance was the MexXY EP. MexAB-OprM was either over- or under-expressed in clinical strains. *P. aeruginosa* strains isolated from Amiens hospital environment did not frequently display a high-level resistance to ciprofloxacin. However, the low-level resistance witnessed for some strains could not be linked with an overexpression of RND EPs. Indeed, RND EPs were significantly underexpressed in nearly all of the 30 non-clinical strains tested in this work. In contrast with what was previously described in carbapenem-resistant strains, a positive correlation between the expression of *oprD* and efflux pump genes *mexB* and *mexF* was witnessed in both CIP-R clinical environmental *P. aeruginosa* strains. A correlation limited to *mexY* expression was nevertheless witnessed for CIP MICs in the statistical analysis gathering the 63 clinical and environmental strains. However, no link with a specific EP component could be ascribed for the magnitude of the reduction in MICs generated by PAβN in the clinical strains. Therefore, no direct quantitative correlation can be made between this phenotypic trait and the genotypic overexpression of one or another of the three RND EPs tested here.

## Author Contributions

CS, BB, SG, and CM performed the experiments. SG and CM analyzed data. AT-M and CM contributed strains. CM conceived and supervised the experiments and wrote the manuscript.

## Conflict of Interest Statement

The authors declare that the research was conducted in the absence of any commercial or financial relationships that could be construed as a potential conflict of interest.

## Footnotes

1 http://bioinfo.ut.ee/primer3/

2 http://vassarstats.net/

## References

[B1] Alav I. Sutton J. M. Rahman K. M. (2018). Role of bacterial efflux pumps in biofilm formation. *J. Antimicrob. Chemother.* 73 2003–2020. 10.1093/jac/dky042 29506149

[B2] Blair J. M. Piddock L. J. (2009). Structure, function and inhibition of RND efflux pumps in Gram-negative bacteria: an update. *Curr. Opin. Microbiol.* 12 512–519. 10.1016/j.mib.2009.07.003 19664953

[B3] Bonomo R. A. Szabo D. (2006). Mechanisms of multidrug resistance in Acinetobacter species and *Pseudomonas aeruginosa*. *Clin. Infect. Dis.* 43 S49–S56. 10.1086/504477 16894515

[B4] Botzenhart K. Döring G. (1993). “Ecology and epidemiology of *Pseudomonas aeruginosa*,” in *Pseudomonas aeruginosa* as an opportunistic pathogen, eds Campa M. Bendinelli M. Friedman H. (New York, NY: Plenum Press), 1–18.

[B5] Braz V. S. Furlan J. P. Fernandes A. F. Stehling E. G. (2016). Mutations in NalC induce MexAB-OprM overexpression resulting in high level of aztreonam resistance in environmental isolates of *Pseudomonas aeruginosa*. *FEMS Microbiol. Lett.* 363:fnw166. 10.1093/femsle/fnw166 27412168

[B6] Bruchmann S. Dötsch A. Nouri B. Chaberny I. F. Häussler S. (2013). Quantitative contributions of target alteration and decreased drug accumulation to *Pseudomonas aeruginosa* fluoroquinolone resistance. *Antimicrob. Agents Chemother.* 57 1361–1368. 10.1128/AAC.01581-12 23274661 PMC3591863

[B7] CLSI. (2015). *Methods for Dilution Antimicrobial Susceptibility Tests for Bacteria That Grow Aerobically; Approved Standard CLSI document M07-A10*, 10th Edn, Wayne, PA: Clinical and Laboratory Standards Institute.

[B8] Del Barrio-Tofiño E. López-Causapé C. Cabot G. Rivera A. Benito N. Segura C. (2017). Genomics and susceptibility profiles of extensively drug-resistant *Pseudomonas aeruginosa* isolates from Spain. *Antimicrob. Agents Chemother.* 61:e1589-17. 10.1128/AAC.01589-17 28874376 PMC5655108

[B9] El-Shaer S. Shaaban M. Barwa R. Hassan R. (2016). Control of quorum sensing and virulence factors of *Pseudomonas aeruginosa* using phenylalanine arginyl β-naphthylamide. *J. Med. Microbiol.* 65 1194–1204. 10.1099/jmm.0.000327 27498852

[B10] EN ISO 16266:2008 (2008). *Water Quality- Detection and Enumeration of Pseudomonas aeruginosa. Method by Membrane Filtration.* Geneva: ISO.

[B11] European Committee on Antimicrobial Susceptibility Testing [EUCAST] (2018). *Breakpoint Tables for Interpretation of MICs and Zone Diameters. Version 8.1, 2018.* Available at: http://www.eucast.org [accessed June 11, 2018].

[B12] Fang X. Fang Z. Zhao J. Zou Y. Li T. Wang J. (2012). Draft genome sequence of *Pseudomonas aeruginosa* strain ATCC 27853. *J. Bacteriol.* 194:3755. 10.1128/JB.00690-12 22740676 PMC3393497

[B13] Gellatly S. L. Hancock R. E. (2013). *Pseudomonas aeruginosa*: new insights into pathogenesis and host defenses. *Pathog. Dis.* 67 159–173. 10.1111/2049-632X.12033 23620179

[B14] Hocquet D. Roussel-Delvallez M. Cavallo J. D. Plésiat P. (2007). MexAB-OprM and MexXY-overproducing mutants are very prevalent among clinical strains of *Pseudomonas aeruginosa* with reduced susceptibility to ticarcillin. *Antimicrob. Agents Chemother.* 51 1582–1583. 10.1128/AAC.01334-06 17220417 PMC1855456

[B15] Horna G. López M. Guerra H. Saénz Y. Ruiz J. (2018). Interplay between MexAB-OprM and MexEF-OprN in clinical isolates of *Pseudomonas aeruginosa*. *Sci. Rep.* 8:16463. 10.1038/s41598-018-34694-z 30405166 PMC6220265

[B16] Ikonomidis A. Tsakris A. Kantzanou M. Spanakis N. Maniatis A. N. Pournaras S. (2008). Efflux system overexpression and decreased OprD contribute to the carbapenem heterogeneity in *Pseudomonas aeruginosa*. *FEMS Microbiol. Lett.* 279 36–39. 10.1111/j.1574-6968.2007.00997.x 18070070

[B17] Köhler T. Epp S. F. Curty L. K. Pechère J. C. (1999). Characterization of MexT, the regulator of the MexE-MexF-OprN multidrug efflux system of *Pseudomonas aeruginosa*. *J. Bacteriol.* 181 6300–6305. 10515918 10.1128/jb.181.20.6300-6305.1999 PMC103763

[B18] Köhler T. Michea-Hamzehpour M. Plésiat P. Kahr A. L. Pechere J. C. (1997). Differential selection of multidrug efflux systems by quinolones in *Pseudomonas aeruginosa*. *Antimicrob. Agents Chemother.* 41 2540–2543. 10.1128/AAC.41.11.2540 9371363 PMC164158

[B19] Lamers R. P. Cavallari J. F. Burrows L. L. (2013). The efflux inhibitor phenylalanine-arginine beta-naphthylamide (PA(N) permeabilizes the outer membrane of Gram-negative bacteria. *PLoS One* 8:e60666. 10.1371/journal.pone.0060666 23544160 PMC3609863

[B20] Larionov A. Krause A. Miller W. (2005). A standard curve based method for relative real time PCR data processing. *BMC Bioinformatics* 6:62. 10.1186/1471-2105-6-62 15780134 PMC1274258

[B21] Lee J. Y. Ko K. S. (2012). OprD mutations and inactivation, expression of efflux pumps and AmpC, and metallo-β-lactamases in carbapenem-resistant *Pseudomonas aeruginosa* isolates from South Korea. *Int. J. Antimicrob. Agents* 40 168–172. 10.1016/j.ijantimicag.2012.04.004 22633564

[B22] Llanes C. Hocquet D. Vogne C. Benali-Baitich D. Neuwirth C. Plésiat P. (2004). Clinical strains of *Pseudomonas aeruginosa* overproducing MexAB-OprM and MexXY efflux pumps simultaneously. *Antimicrob. Agents Chemother.* 48 1797–1802. 10.1128/AAC.48.5.1797-1802.2004 15105137 PMC400543

[B23] Lomovskaya O. Warren M. S. Lee A. Galazzo J. Fronko R. Lee M. (2001). Identification and characterization of inhibitors of multidrug resistance efflux pumps in *Pseudomonas aeruginosa*: novel agents for combination therapy. *Antimicrob. Agents Chemother.* 45 105–116. 10.1128/AAC.45.1.105-116.2001 11120952 PMC90247

[B24] López-Causapé C. Cabot G. Del Barrio-Tofiño E. Oliver A. (2018). The versatile mutational resistome of *Pseudomonas aeruginosa*. *Front. Microbiol.* 9:685. 10.3389/fmicb.2018.00685 29681898 PMC5897538

[B25] Mao W. Warren M. S. Lee A. Mistry A. Lomovskaya O. (2001). MexXY-OprM efflux pump is required for antagonism of aminoglycosides by divalent cations in *Pseudomonas aeruginosa*. *Antimicrob. Agents Chemother.* 45 2001–2007. 10.1128/AAC.45.7.2001-2007.2001 11408215 PMC90592

[B26] Maravić A. Šamanić I. Šprung M. Fredotović Z. Ilić N. Dragičević J. (2018). Broad-spectrum resistance of *Pseudomonas aeruginosa* from shellfish: infrequent acquisition of novel resistance mechanisms. *Environ. Monit. Assess.* 190:81. 10.1007/s10661-018-6471-3 29335824

[B27] Maseda H. Sawada I. Saito K. Uchiyama H. Nakae T. Nomura N. (2004). Enhancement of the mexAB-oprM efflux pump expression by a quorum-sensing autoinducer and its cancellation by a regulator, MexT, of the mexEF-oprN efflux pump operon in *Pseudomonas aeruginosa*. *Antimicrob. Agents Chemother.* 48 1320–1328. 10.1128/AAC.48.4.1320-1328.2004 15047536 PMC375252

[B28] Mesaros N. Glupczynski Y. Avrain L. Caceres N. E. Tulkens P. M. Van Bambeke F. (2007). A combined phenotypic and genotypic method for the detection of Mex efflux pumps in *Pseudomonas aeruginosa*. *J. Antimicrob. Chemother.* 59 378–386. 10.1093/jac/dkl504 17289770

[B29] Minagawa S. Inami H. Kato T. Sawada S. Yasuki T. Miyairi S. (2012). RND type efflux pump system MexAB-OprM of *Pseudomonas aeruginosa* selects bacterial languages, 3-oxo-acyl-homoserine lactones, for cell-to-cell communication. *BMC Microbiol.* 12:70. 10.1186/1471-2180-12-70 22574700 PMC3460771

[B30] Morita Y. Komori Y. Mima T. Kuroda T. Mizushima T. Tsuchiya T. (2001). Construction of a series of mutants lacking all of the four major mex operons for multidrug efflux pumps or possessing each one of the operons from *Pseudomonas aeruginosa* PAO1: MexCD-OprJ is an inducible pump. *FEMS Microbiol. Lett.* 202 139–143. 10.1111/j.1574-6968.2001.tb10794.x 11506922

[B31] Morita Y. Tomida J. Kawamura Y. (2012). MexXY multidrug efflux system of *Pseudomonas aeruginosa*. *Front. Microbiol.* 3:408 10.3389/fmicb.2012.00408 PMC3516279 23233851

[B32] Piddock L. J. V. (2006). Clinically relevant chromosomally encoded multidrug resistance efflux pumps in bacteria. *Clin. Microbiol. Rev.* 19 382–402. 10.1128/CMR.19.2.382-402.2006 16614254 PMC1471989

[B33] Poole K. (2005). Efflux-mediated antimicrobial resistance. *J. Antimicrob. Chemother.* 56 20–51. 10.1093/jac/dki171 15914491

[B34] Poole K. (2011). *Pseudomonas aeruginosa*: resistance to the max. *Front. Microbiol.* 2:65. 10.3389/fmicb.2011.00065 21747788 PMC3128976

[B35] Poole K. Krebes K. McNally C. Neshat S. (1993). Multiple antibiotic resistance in *Pseudomonas aeruginosa*: evidence for involvement of an efflux operon. *J. Bacteriol.* 175 7363–7372. 10.1128/jb.175.22.7363-7372.1993 8226684 PMC206881

[B36] Rehman A. Patrick W. M. Lamont I. L. (2019). Mechanisms of ciprofloxacin resistance in *Pseudomonas aeruginosa*: new approaches to an old problem. *J. Med. Microbiol.* 68 1–10. 10.1099/jmm.0.000873 30605076

[B37] Sonnet P. Izard D. Mullié C. (2012). Prevalence of efflux-mediated ciprofloxacin and levofloxacin resistance in recent clinical isolates of *Pseudomonas aeruginosa* and its reversal by the efflux pump inhibitors 1-(1-naphthylmethyl)-piperazine and phenylalanine-arginine-β-naphthylamide. *Int. J. Antimicrob. Agents* 39 77–80. 10.1016/j.ijantimicag.2011.08.005 21974858

[B38] Tomás M. Doumith M. Warner M. Turton J. F. Beceiro A. Bou G. (2010). Efflux pumps, OprD porin, AmpC beta-lactamase, and multiresistance in *Pseudomonas aeruginosa* isolates from cystic fibrosis patients. *Antimicrob. Agents Chemother.* 54 2219–2224. 10.1128/AAC.00816-09 20194693 PMC2863613

[B39] Uwate M. Ichise Y. K. Shirai A. Omasa T. Nakae T. Maseda H. (2013). Two routes of MexS-MexT-mediated regulation of MexEF-OprN and MexAB-OprM efflux pump expression in *Pseudomonas aeruginosa*. *Microbiol. Immunol.* 57 263–272. 10.1111/1348-0421.12032 23586630

[B40] Vatcheva-Dobrevska R. Mulet X. Ivanov I. Zamorano L. Dobreva E. Velinov T. (2013). Molecular epidemiology and multidrug resistance mechanisms of *Pseudomonas aeruginosa* isolates from Bulgarian hospitals. *Microb. Drug Resist.* 19 355–361. 10.1089/mdr.2013.0004 23600605

[B41] Vila J. Martínez J. L. (2008). Clinical impact of the over-expression of efflux pump in nonfermentative Gram-negative bacilli, development of efflux pump inhibitors. *Curr. Drug Targets* 9 797–807. 10.2174/138945008785747806 18781925

[B42] Xavier D. E. Picao R. C. Girardello R. Fehlberg L. C. C. Gales A. C. (2010). Efflux pumps expression and its association with porin down-regulation and (-lactamase production among *Pseudomonas aeruginosa* causing bloodstream infections in Brazil. *BMC Microbiol.* 10:217. 10.1186/1471-2180-10-217 20704733 PMC2927533

[B43] Xia J. Gao J. Tang W. (2016). Nosocomial infection and its molecular mechanisms of antibiotic resistance. *Biosci. Trends* 10 14–21. 10.5582/bst.2016.01020 26877142

[B44] Yoneda K. Chikumi H. Murata T. Gotoh N. Yamamoto H. Fujiwara H. (2005). Measurement of *Pseudomonas aeruginosa* multidrug efflux pumps by quantitative real-time polymerase reaction. *FEMS Microbiol. Lett.* 243 125–131. 10.1016/j.femsle.2004.11.048 15668010

