# Supplementary material for: MexXY Multidrug Efflux System Is More Frequently Overexpressed in Ciprofloxacin Resistant French Clinical Isolates Compared to Hospital Environment Ones
Source: Front Microbiol. 2019 Feb 26;10:366. doi: 10.3389/fmicb.2019.00366 (PMC6399115; doi:10.3389/fmicb.2019.00366)
Supplement: Supplementary file 1 [file Table_1.docx]

Supplementary Material

Resistance-Nodulation-Division efflux pumps in *Pseudomonas aeruginosa*: comparison of their expression in environmental and clinical strains.

Cristiano Serra, Bakhta Bouharkat, Aicha Tir Touil-Meddah, Stéphanie Guénin, Catherine Mullié^*^

*** Correspondence:** Catherine Mullié: [catherine.mullie@u-picardie.fr](mailto:catherine.mullie@u-picardie.fr)

Table S1. Individual Normalized Calibrated Ratios (mean ± standard error of the mean). Normalization was performed using results obtained for Pseudomonas aeruginosa AM19, a clinical strain susceptible to ciprofloxacin.

|  | *mexB* | *mexF* | *mexY* | *oprD* |
| --- | --- | --- | --- | --- |
| Collection strain |  |  |  |  |
| DSM1117 | 7.3 ± 1.24^*^ | 1.0 ± 0.122 | 0.25 ± 0.036^†^ | ND |
|  |  |  |  |  |
| Clinical strains |  |  |  |  |
| AM1 | 0.8 ± 0.04 | 0.2 ± 0.01^†^ | 0.6 ± 0.03 | ND ^a^ |
| AM3 | 10 ± 0.49^*^ | 0.98 ± 0.159 | 0.74 ± 0.106 | ND |
| AM10 | 1 ± 0.05 | 0.2 ± 0.01^†^ | 2.8 ± 0.51^*^ | ND |
| AM13 | 0.8 ± 0.24 | 0.7 ± 0.02 | 3.8 ± 1.00 | ND |
| AM17 | 1.1 ± 0.03 | 3.8 ± 0.14^*^ | 1.3 ± 0.04 | 1.3 ± 0.04 |
| AM27 | 2.1 ± 0.07 | 6.6 ± 0.23^*^ | 3.8 ± 0.10^*^ | 1.2 ± 0.04 |
| AM32 | 1.4 ± 0.05 | 1.9 ± 0.07 | 0.77 ± 0.019 | 0.74 ± 0.021 |
| AM33 | 3.7 ± 0.28^*^ | 1.2 ± 0.1 | 0.51 ± 0.039 | 2.2 ± 0.17 |
| AM42 | 1.8 ± 0.05 | 5 ± 0.18^*^ | 10 ± 0.2^*^ | 3 10^-4^ ± 1 10^-5†^ |
| AM44 | 0.002 ± 0.0004^†^ | 0.02 ± 0.003^†^ | 9.7 ± 2.25^*^ | ND |
| AM50 | 0.003 ± 0.0009^†^ | 0.15 ± 0.009^†^ | 16 ± 4.0^*^ | ND |
| AM52 | 0.07 ± 0.017^†^ | 0.01 ± 0.001^†^ | 17 ± 3.7^*^ | ND |
| AM56 | 7.7 ± 1.15^*^ | 2.1 ± 0.45 | 0.24 ± 0.073^†^ | 3 10^-4^ ± 5 10^-5†^ |
| AM58 | 0.13 ± 0.023^†^ | 0.005 ± 0.0008^†^ | 25 ±6.4^*^ | ND |
| AM 60 | 5.8 ± 0.31^*^ | 0.59 ± 0.031 | 1.05 ± 0.188 | 1.1 ± 0.06 |
| AM66 | 0.48 ± 0.126 | 0.13 ± 0.017^†^ | 0.40 ± 0.05 | ND |
| AM69 | 0.10 ± 0.028^†^ | 0.20 ± 0.033^†^ | 9.4 ± 2.05^*^ | ND |
| AM74 | 3.3 ± 0.41^*^ | 0.56 ± 0.076 | 4.6 ± 0.73^*^ | ND |
| AM83 | 0.58 ± 0.162 | 0.11 ± 0.022^†^ | 7.5 ± 1.56^*^ | 1.5 ± 0.16 |
| AM85 | 13 ± 0.8^*^ | 13 ± 0.94^*^ | 15 ± 0.1^*^ | 2.3 ± 0.37 |
| AM86 | 5.3 ± 0.73^*^ | 2.7 ± 0.26 | 4.3 ± 0.47 | 1.3 ± 0.38 |
| AM88 | 0.004 ± 0.0002^†^ | 0.03 ± 0.003^†^ | 19 ± 1.0^*^ | ND |
| AM99 | 0.11 ± 0.010^†^ | 0.02 ± 0.001^†^ | 41 ± 3.8^*^ | ND |
| AM100 | 0.48 ± 0.126 | 0.13 ± 0.017^†^ | 0.40 ± 0.055 | ND |
| AM110 | 0.11 ± 0.028^†^ | 0.20 ± 0.034^†^ | 9.4 ± 2.05^*^ | ND |
| AM113 | 0.58 ± 0.162 | 0.11 ± 0.022^†^ | 7.5 ± 1.56^*^ | ND |
| AM115 | 10 ± 2.5^*^ | 0.85 ± 0.115 | 2.8 ± 0.69 | 1.3 ± 0.38 |
| AM126 | 8.1 ± 2.6^*^ | 2.9 ± 1.09 | 0.9 ± 0.25 | 0.001 ± 0.0005^†^ |
| AM127 | 4.5 ± 1.68 | 1.2 ± 0.58 | 0.61 ± 0.23 | 1.3 ± 0.44 |
| AM128 | 0.1 ± 0.01^†^ | 0.02 ± 0.005^†^ | 0.02 ± 0.003^†^ | ND |
| AM129 | 5.9 ± 2.28 | 0.8 ± 0.38 | 0.7 ± 0.31 | 1.3 ± 0.51 |
| AM130 | 0.1 ± 0.02^†^ | 0.05 ± 0.013^†^ | 0.06 ± 0.009^†^ | ND |
| AM131 | 0.004 ± 0.0001^†^ | ND | 0.007 ± 0.0011^†^ | 0.1 ± 0.02^†^ |
|  |  |  |  |  |
| Non-clinical strains |  |  |  |  |
| ENV1 | ND | 5 10^-4^ ± 9 10^-5†^ | 1 10^-4^ ± 2 10^-5†^ | ND |
| ENV2 | 0.7± 0.29 | 0.2 ± 0.08^†^ | 0.2 ± 0.06^†^ | 0.9 ± 0.32 |
| ENV3 | 0.9 ± 0.24 | 0.5 ± 0.19 | 0.06 ± 0.018^†^ | 2.3 ± 0.84 |
| ENV4 | 0.1 ± 0.02^†^ | 0.004 ± 0.0004^†^ | 0.002 ± 0.0002^†^ | ND |
| ENV5 | 1 10^-5^ ± 5 10^-5†^ | 0.3 ± 0.10^†^ | 0.2 ± 0.07^†^ | ND |
| ENV6 | 0.2 ± 0.07^†^ | 0.7 ± 0.24 | 0.1 ± 0.04^†^ | 3 10^-4^ ± 1 10^-4†^ |
| ENV7 | 0.001 ± 0.0004^†^ | 4 10^-5^ ± 9 10^-6†^ | 5 10^-5^ ± 1 10^-5†^ | ND |
| ENV8 | 0.001 ± 0.0002^†^ | 0.0003 ± 6 10^-5†^ | 0.0003 ± 5 10^-5†^ | ND |
| ENV9 | 4 10^-4^ ± 6 10^-5†^ | 8 10^-5^ ± 2 10^-5†^ | 5 10^-5^ ± 1 10^-5†^ | ND |
| ENV10 | ND | ND | ND | ND |
| ENV11 | 0.009 ± 0.0017^†^ | 8 10^-5^ ± 1 10^-5†^ | 1 10^-4^± 2 10^-5†^ | ND |
| ENV12 | 0.008 ± 0.0018^†^ | 6 10^-5^ ± 1 10^-5†^ | 1 10^-5^ ± 3 10^-6†^ | 0.009 ± 0.0021^†^ |
| ENV13 | 0.01 ± 0.003^†^ | 1 10^-4^ ± 3 10^-5†^ | ND | 9 10^-5^ ± 1 10^-5†^ |
| ENV14 | 0.03 ± 0.007^†^ | ND | 1 10^-6^ ± 2 10^-7†^ | 0.01 ± 0.002^†^ |
| ENV15 | 0.2 ± 0.03^†^ | 0.002 ± 0.0006^†^ | 0.002 ± 0.0004^†^ | 0.1 ± 0.02^†^ |
| ENV16 | 0.2 ± 0.07^†^ | ND | ND | 0.2 ± 0.04^†^ |
| ENV17 | 0.01 ± 0.002^†^ | ND | 0.004 ± 0.0013^†^ | 0.3 ± 0.04^†^ |
| ENV18 | 0.07 ± 0.015^†^ | 1 10^-4^ ± 4 10^-5†^ | 0.0002 ± 3 10^-5†^ | 0.2 ± 0.02^†^ |
| E NV19 | 0.07 ± 0.018^†^ | 6 10^-4^ ± 1.7 10^-4†^ | 4 10^-4^ ± 1.1 10^-4†^ | 0.1 ± 0.018^†^ |
| ENV20 | 0.06 ± 0.011^†^ | 2 10^-4^ ± 5 10^-5†^ | 2 10^-4^ ± 3 10^-5†^ | 0.1 ± 0.018^†^ |
| ENV21 | 0.04 ± 0.013^†^ | ND | ND | 0.08 ±0.019^†^ |
| ENV22 | 0.05 ± 0.009^†^ | ND | 0.001 ± 0.0002^†^ | 0.04 ± 0.005^†^ |
| ENV23 | ND | ND | 0.002 ± 0.0004^†^ | ND |
| ENV24 | ND | ND | 5 10^-4^ ±7 10^-5†^ | ND |
| ENV25 | 0.1 ± 0.02^†^ | 0.005 ± 0.0011^†^ | 5 10^-4^ ± 7 10^-5†^ | ND |
| ENV26 | ND | ND | ND | ND |
| ENV27 | ND | ND | ND | ND |
| ENV28 | ND | 0.04 ± 0.01^†^ | 0.01 ± 0.005^†^ | ND |
| ENV29 | ND | 0.02 ± 0.067^†^ | 0.05 ± 0.014^†^ | ND |
| ENV30 | ND | 0.02 ± 0.005^†^ | 0.007 ± 0.0027^†^ | ND |

^a^: Not Detected

^*^: significant overexpression (Single sample t-test vs. a theoretical mean of 2, p<0.05)

^†^: significant underexpression (Single sample t-test vs. a theoretical mean of 0.5, p<0.05)

**
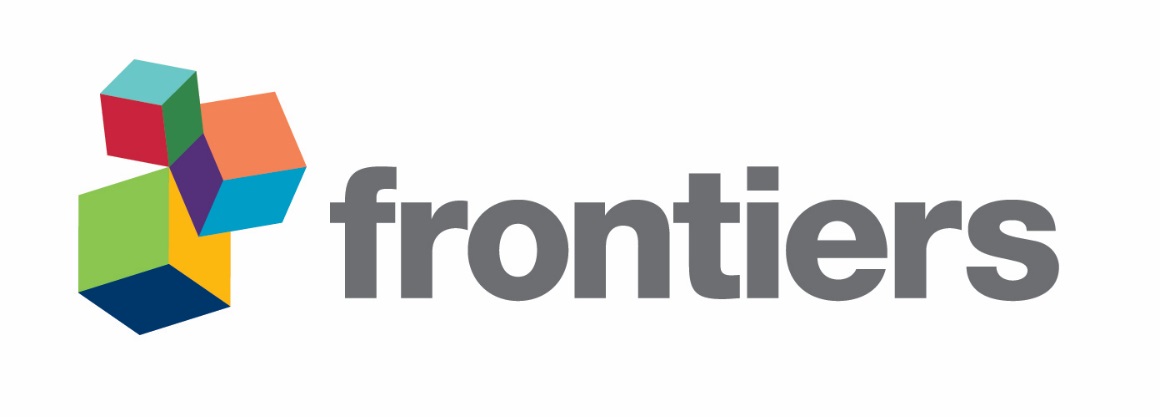
**
